# Pounds on Pressure: Impact of Weight Loss on Intracranial Pressure in Idiopathic Intracranial Hypertension

**DOI:** 10.51894/001c.164374

**Published:** 2026-07-31

**Authors:** Anika Tahmeed, Khabbab Alnimer, Abdallah Almawazreh, Kamal Khalil

**Affiliations:** 1 Detroit Medical Center- Sinai Grace Hospital

## 39

### Background

Idiopathic intracranial hypertension (IIH) is characterized by elevated intracranial pressure (ICP) without an identifiable structural cause and predominantly affects young women with obesity. Increasing obesity prevalence parallels a rising incidence of IIH, contributing to substantial morbidity, including chronic headache and irreversible visual loss. Although weight reduction is a cornerstone of IIH management, the magnitude of weight loss required to achieve clinically meaningful ICP reduction remains incompletely defined.

### Objectives

To evaluate the association between degree of weight loss and reduction in intracranial pressure in adults with IIH, regardless of intervention type.

### Methods

A systematic review of the literature was conducted using PubMed, focusing on human clinical studies published within the past five years. Eligible studies included adults (≥19 years) with confirmed IIH and baseline BMI ≥30 kg/m² who underwent weight loss interventions with reported ICP-related outcomes. Study designs included randomized controlled trials, observational cohorts, and retrospective analyses. Pediatric, pregnant, non-clinical, and non–free PubMed studies were excluded. After title, abstract, and full-text screening, four studies met inclusion criteria.

### Results

Across included studies, greater weight loss was consistently associated with improved IIH outcomes. A randomized clinical trial comparing bariatric surgery with community-based weight management demonstrated significantly greater weight loss in the surgical group (−26.6 kg at 24 months), accompanied by substantial ICP reduction (−8.2 cm CSF) and improved quality of life. Normalization of ICP (≤25 cm CSF) required approximately 24% loss of baseline body weight in women with BMI ≥35 kg/m². A large retrospective cohort study using the TriNetX database showed greater improvement in papilledema, headache burden, visual field defects, and reduced need for acetazolamide and optic nerve sheath fenestration among bariatric surgery patients, though direct ICP measurements were inconsistently reported. Additionally, a randomized trial of the GLP-1 receptor agonist exenatide demonstrated short-term ICP reduction without cognitive impairment, suggesting a potential weight-independent mechanism; however, long-term efficacy remains uncertain.

### Conclusion

Weight loss plays a central role in reducing ICP and improving clinical outcomes in IIH, with bariatric surgery providing the most consistent and sustained benefit in severe obesity. Further large-scale studies are needed to clarify the long-term role of pharmacologic therapies.

